# Impact of an Innovative Educational Strategy on Medication Appropriate Use and Length of Stay in Elderly Patients

**DOI:** 10.1097/MD.0000000000000918

**Published:** 2015-06-19

**Authors:** Graziamaria Corbi, Giovanni Gambassi, Gennaro Pagano, Giusy Russomanno, Valeria Conti, Giuseppe Rengo, Dario Leosco, Roberto Bernabei, Amelia Filippelli, Nicola Ferrara

**Affiliations:** From Department of Medicine and Health Sciences, University of Molise, Campobasso, Italy (GC, GP); Centro Medicina dell’Invecchiamento, Università Cattolica del Sacro Cuore, Rome, Italy (GG, RB); Division of Brain Sciences, Imperial College London, UK (GP); Department of Medicine and Surgery, University of Salerno, Baronissi (SA), Italy (GR, VC, AF); Department of Translational Medical Sciences, Federico II University of Naples, Naples, Italy (GR, DL, NF); and Salvatore Maugeri Foundation, IRCCS, Scientific Institute of Telese, Telese Terme (BN), Italy (GR, NF).

## Abstract

To evaluate the impact of an educational strategy on potentially inappropriate medications (PIMs) and length of stay in hospitalized elderly patients.

**Design:**

An open study, with two cross-sectional surveys interspersed with an educational program (PRE phase and POST phase), has been performed in order to compare the PIMs number before and after the introduction of an educational strategy. The study included 2 phases: PRE, in which patients were enrolled as control group; POST, in which an educational strategy on the PIMs use was introduced among physicians, and patients were enrolled as intervention group.

**Setting:**

Italian residential rehabilitation Centre.

Inclusion criteria were ≥2 active chronic diseases and the current use of ≥4 medications.

The educational strategy consisted of a 3-day course on strategies to prevent PIMs and a computerized tool running on a Personal Digital Assistant (PDA) device to check for PIMs.

**Outcomes:**

The primary was the PIMs number, the secondary the length of stay.

**Results:**

A total of 790 patients, 450 controls and 340 cases, were enrolled. According to the Beers criteria, 52.3% of the study population received ≥1 PIMs, 18.73% ≥2, and 2.4% ≥4 PIMs. A significant reduction of PIMs (*P* = 0.020) and length of stay (*P* < 0.0001) were seen in the intervention group. At multivariate analysis, PIMs significantly correlated with age, drugs number, and the intervention, and the length of stay significantly correlated with disease count, comorbidities, and intervention.

These data suggest that our educative instrument may be useful in reducing the PIMs number and length of hospitalization in elderly with a high number of drugs and comorbidities.

## INTRODUCTION

Appropriateness indicates that the potential benefits of a medication outweigh the potential risks and represents a crucial point for the choice of the best drug. Inappropriate medications can be defined as “medications or medication classes that should generally be avoided in persons 65 years or older because they are either ineffective or they pose unnecessarily high risk for older persons and a safer alternative is available.”^1^ Many elderly patients routinely receive potentially inappropriate medications (PIMs), such as sedative-hypnotics, analgesics, histamine blockers, antibiotics, laxatives that can cause harm, directly or through interactions.^2–6^

The prevalence of inappropriateness increases with the age reaching a 70% in a geriatric hospital setting.^7^ PIMs are common in older patients for the physiological changes related to the age and for a greater degree of frailty and multiple coexisting conditions. In turn, exposure to inappropriate therapy is associated with an increase in terms of adverse events,^8^ morbidity, mortality, and health costs.^9,10^ Several different tools^1,11^ have measured inappropriateness and specific criteria have been formulated to identify PIMs.^1,11–13^ However, the applicability and effects in clinical practice for these various measures remains uncertain.^14,15^

Several studies have been conducted on this topic with inconclusive results. Computer data entry and feedback procedures, which have been shown to decrease polypharmacy and drug–drug interactions,^16^ visual identification of medicines, continuous medication review and thorough patient education to optimize polypharmacy,^17^ computerized decision support,^18^ multidisciplinary case conferences^19^ represent only some kind of tools used to reduce PIMs. However, conclusive evidence is currently lacking, probably because there were heterogeneities in settings, study population and evaluation methods, and the optimal method to avoid inappropriate medication is at present unknown. The aim of this study was to evaluate the impact of an educational strategy on PIMs incidence in elderly patients 65 years and older admitted postacutely to a residential rehabilitation medical centre in Italy.

## MATERIALS AND METHODS

Patients’ information and consent forms were approved by the Ethical committee of the coordinating Centre at the University of Molise. The study was conducted in accordance with the World Medical Association's 2008 Declaration of Helsinki. This report adheres to the consolidated standards for the reporting of observational trials and was written according to the STROBE guidelines for Observational Studies in Epidemiology.^20^

### Study Design

We conducted an open study, with 2 cross-sectional surveys interspersed with an educational program (PRE phase and POST phase),^21^ in order to compare patients PRE (control group) and POST (intervention group) the introduction of an educational/informative strategy in term of number of PIMs and of length of stay. The study included 2 different phases. In an initial phase (PRE) lasting 4 months all patients consecutively admitted to the rehabilitation units participating to the study were enrolled and followed up until discharge. After this initial phase, an educational/informative strategy on the use of inappropriate medications was introduced and during a second 4 months period (POST) all consecutive patients admitted to the participating units were recruited in a similar fashion as phase one. The number of PIMs was evaluated at the discharge, using a specific software (Farmadati library, http://www.farmadati.it).

### Population

Patients 65 years and older were enrolled in the study at admission to the rehabilitation unit. Inclusion criteria were the presence of 2 or more active chronic medical conditions (cardiovascular-respiratory system, gastrointestinal system, genitourinary system, muscle-skeletal system, neuropsychiatric system, and general system) and the current use of 4 or more concomitant medications. The postacute intensive rehabilitation Centre admitted patients discharged from hospitals for acute cardiovascular and neurological accidents. All patients were treated with the therapy received at the moment of the hospital's discharge.

### Intervention

The education strategy consisted of a 3-day intensive training course and of a computerized tool for physicians. The education course, provided by specialists on inappropriate prescriptions, was a training module on epidemiology, pathogenesis and strategies to prevent drug inappropriateness, interactions and Adverse Drug Reactions (ADRs) including the principles of geriatric pharmacology, the disease itself, and factors that can lead to complications.

The specifically qualifying elements of the training were appropriately selected based also on an evaluation of the common problematic areas of elderly patients and based also on information gathered in the patient group recruited in the control period. Educational leaflets were prepared in the form of a self-study program that includes information on the previously mentioned topics and after every educational session they were given to the physicians. The authors developed all the educational materials. The training sessions also included teaching the use of the computerized tool.

The computerized tool worked on a Personal Digital Assistant (PDA) device to check for inappropriateness prior to any new medication prescription. The tool consisted of an information system already embedded in the commercially available Atl@nte system, which consults the Pharmaceutical Handbook for criteria of inappropriateness. The application started by using a special icon installed on the PDA. The system, after authentication with a password, presented a grid with the list of drugs researched and selected, and clickable buttons (drug research, inappropriateness of each drug with detailed information about the drug, deletion of the drug selected, cleaning the grill). The grid for each selected drug contained the following information: Marketing Authorisation code (AIC), Anatomical Therapeutic Chemical (ATC) code, short description of the drug and the active ingredient.

After entering the search criteria by clicking on the “Find” button at the bottom of the screen was shown the list of drugs that met the criteria. Once decided which drug to select by clicking on the button “Select the drug,” the search form closed and again appeared the first screen containing the list of selected drugs. Concerning the inappropriateness, the system showed whether in the drugs list of the grid, there were PIM, and these were highlighted with three different colors: RED (to avoid always), ORANGE (rarely appropriate), YELLOW (some indications). By clicking the “inappropriateness,” the program reported the additional notes related to the selected drug. The PIMs were visualized by the PDA at the moment of the drug prescription in the intervention group, but the physicians could or could not decide to confirm the drug administration. In the intervention group any therapy in all patients was confirmed or modified at admission by using the PDA. The PIMs number was determined at the end of the study for both groups.

### Endpoints

The primary outcomes of the study were the cumulative number of PIMs. Secondary endpoint was the length of stay. For the definition of inappropriateness, we used Beers 2003^12^ criteria. The number of PIMs were monitored through dedicated software implemented using the Farmadati library (http://www.farmadati.it) and evaluated at patient discharge. The system was supported at the Handbook of Pharmaceutical Company Farmadati Italy srl and the system Atl@nte for the period of the project used the drug-index of the company. With regard to the aspects of optimization of therapy, the appropriateness and the best compliance of therapy were referred to all drugs.

### Data Collection

For data collection, we used the centralized Atl@nte system that contains an electronic medical record that allows data entry online. The Atl@ante system is an organizational and management tool, which includes multiple geriatric assessment instruments including the Italian version of the International Residential Assessment Instrument (interRAI). It targets people over 65 years who require needs assessment for access to publicly funded services, and consists of a multidimensional geriatric assessment (VMD) system intended to determine a hospitalized older persons’ medical, psychosocial, and functional capacity and needs. Its objective is to develop an overall plan for treatment and long-term follow-up based on a common set of standardized items that can be used in various care settings. The electronic medical record contains: (a) physical examination and clinical assessment, (b) the Cumulative Illness Rating Scale (CIRS) with reference to conditions classified according to the International Classification of Diseases, 9th revision, Clinical Modification (ICD 9-CM), (c) VMD including the Geriatric Depression Scale and the EURO QoL 5D, (d) medical history including previous tests and examinations, (e) medications prescribed and administered, (f) clinical daily notes with indications of all diagnostic tests, (g) a record about healthcare-related resource use. Training sessions for using the electronic health record were conducted at the onset of data collection and continuous support and a manual explaining the use of the software were provided.

### Statistical Analysis

Continuous variables are expressed as mean ± standard deviation and compared by the use of Student *t* test (normally distributed) or as median ± interquartile range value and compared by the use of Mann–Whitney *U* test (not normally distributed), as appropriate. Normality of data distribution was evaluated using the Kolmogorov-Smirnov test. Not normally distributed continuous variables were natural log transformed. Categorical variables are expressed as proportion and compared by use of χ^2^ test.

Correlation between variables was assessed by linear regression analysis and variables that revealed a statistical significance at univariate model where then included in a multivariate analysis.

To determine the independent predictors of the number of PIMs, linear regression analysis was performed and variables achieving *P* < 0.10 on univariate analysis were then included in a multivariate analysis. To fulfill the assumption of linearity of the not normally distributed variables, they were included in the linear regression analysis as their natural logarithmic function. The same analysis was performed for the length of stay, as dependent variable. All data were collected in an Excel database and analyzed by Statistical Package for Social Science (SPSS) version 19.0 (SPSS, Inc, Chicago, IL). Statistical significance was accepted at *P* < 0.05.

### Sample Size

The sample size was estimated by referring to epidemiological studies and intervention carried out on elderly patients. We expected that the intervention would lead to a reduction of PIMs of at least 10%. Our study was powerful enough to be able to detect with a power of 80% (beta = 20%) to 2-tailed significance level of 5% (alpha 5%), a reduction of PIMs from 65.1% to 55.0%. The relative group size was 57% (n = 450) for the group with highest expected incidence (without intervention) and 43% (n = 340) for the lowest expected incidence (with intervention).

## RESULTS

### Study Patients

A total of 790 patients, 450 during the PRE phase acting as controls and 340 cases during the POST phase after the implementation of the educational/informative strategy were enrolled, representing the intervention group. The main sociodemographic characteristics and drugs number of the 790 subjects are shown in Table 1.

**TABLE 1:**
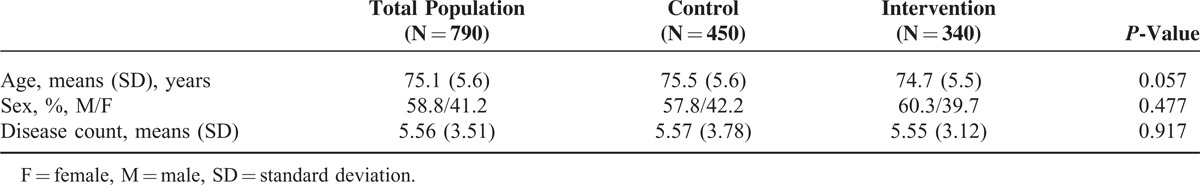
Demographic Characteristics of the Study Population

**FIGURE 1:**
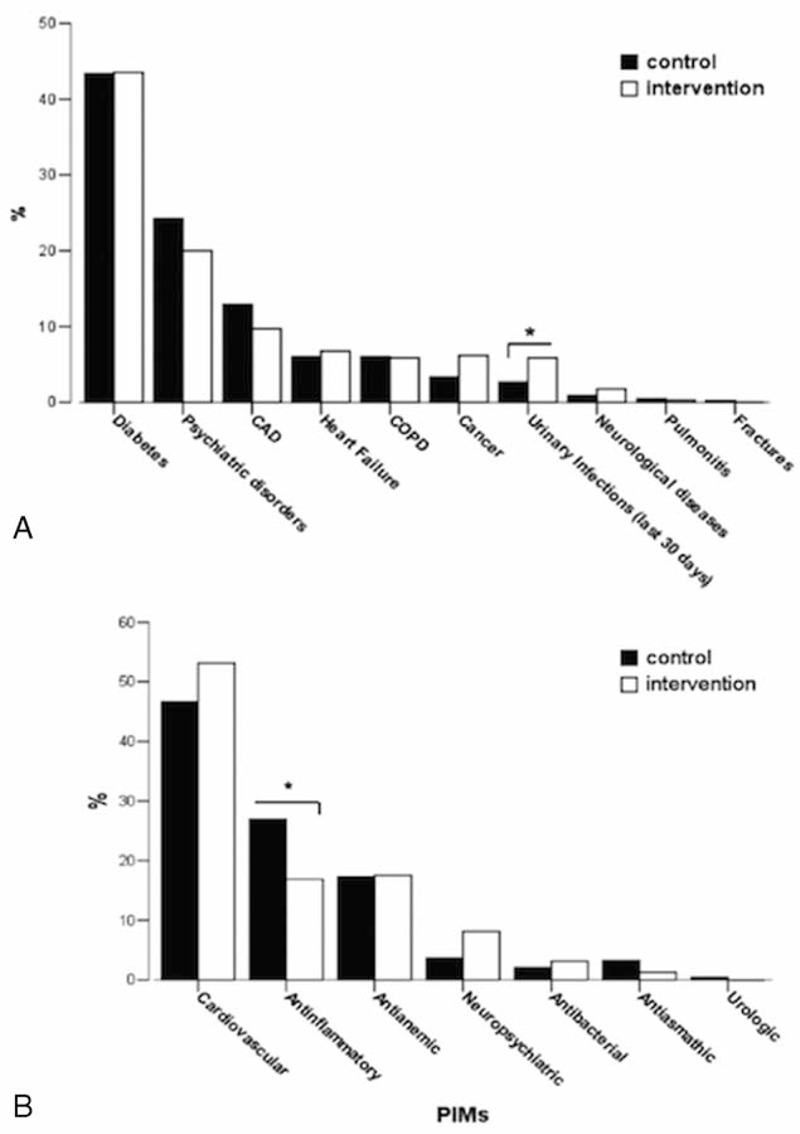
(A) Diseases percentage distribution of study population stratified by control and intervention group. Some differences were found between control and intervention group. In the intervention there were more urinary infections in the last 30 days than in the control group; ^∗^*P* = 0.017. (B) Distribution of the inappropriate drugs for drug category in the study. The most frequent drugs responsible of potentially inappropriate medications (PIMs) were represented by the antiarrhythmics in both groups. The only significant difference was found in the % of PIMs for antinflammatory/antirheumatic drugs; ^∗^*P* = 0.005.

Median number of different drugs prescribed to each individual was 8 (interquartile range [IQR] 7, 10, range 1–22) in the control and 9 (IQR 7, 11, range 1–22) in the intervention group. Although the disease count was similar in the 2 groups, the intervention one was characterized by a significant higher CIRS comorbidity and severity indexes in both 13 and 14 parameters comparing to the controls and some significant differences were also found in the frequency of urinary infections (*P* = 0.017) between the 2 groups (Table 2 and Figure 1A). Nevertheless, a significant difference was reported in the length of stay with an average duration lower in the intervention group compared to the control (*P* < 0.0001; Table 2). In both groups, the cardiovascular molecules represented the main drugs used, with significant difference only in the higher use of antinflammatory and neurological drugs in the intervention in respect to the control group (Table 2).

**TABLE 2:**
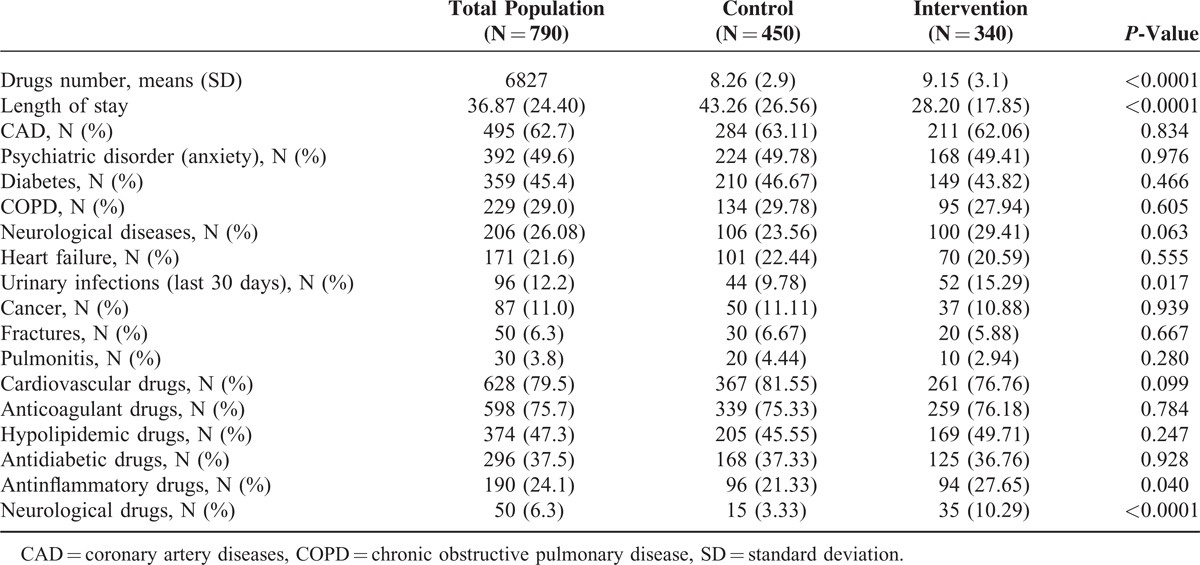
Other Characteristics of the Study Population

### Inappropriate Prescriptions

By using Beers 2003 criteria,^11^ 413 persons ≥65 years (52.3% of the study population) received 1 or more PIMs, 148 (18.73% of the study population) received 2 or more, and 19 people (2.4%) were affected by 4 or more PIMs with a maximum of 7 different indicator hits affecting 1 person (Table 3). The total number of hits was 626. The most frequent drugs responsible of PIMs were represented by antiarrhythmics in both groups. The only difference was in the intervention group a lower incidence of antinflammatory/antirheumatic PIMs (*P* = 0.005; Figure 1B).

**TABLE 3:**
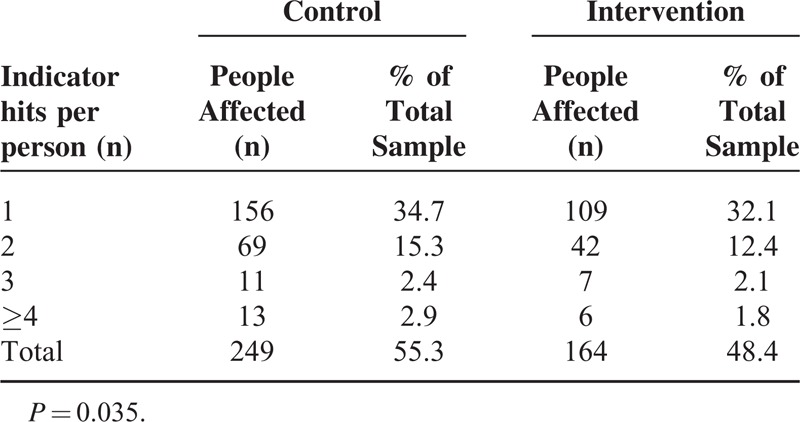
Prevalence of Potentially Inappropriate Medications (PIMs) per Person According to the Beers and Zhan Criteria

**FIGURE 2:**
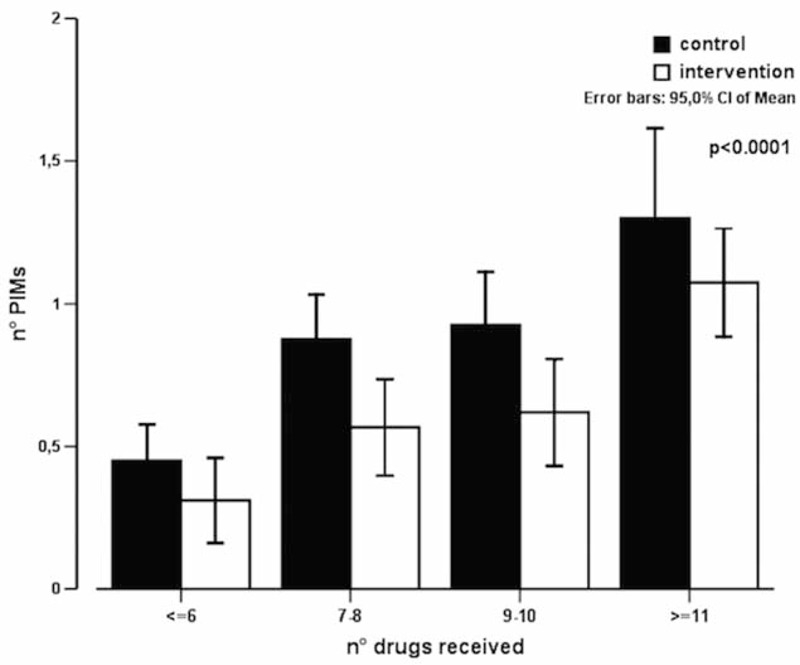
Proportion of patients receiving ≥1 potentially inappropriate medication (PIMs), in relation to the drugs total number.

A significant reduction of PIMs was seen in the intervention group (0.86 (1.05) vs 0.70 (0.89); *P* = 0.020; 95% CI = 0.026–0.305) especially for the category with more than 4 inappropriate prescriptions. The lower number of inappropriate prescriptions was better evident by the lower proportion of patients receiving 1 or more potentially inappropriate medication in relation to the total number of pharmaceutical substances used in the intervention versus the control group (Figure 2; *P* < 0.0001). At multivariate analysis, the predictors of PIMs were age, number of drugs, and the intervention (Table 4). At multivariate analysis, length of stay significantly correlated with disease count, comorbidity index, and the intervention (Table 5).

**TABLE 4:**
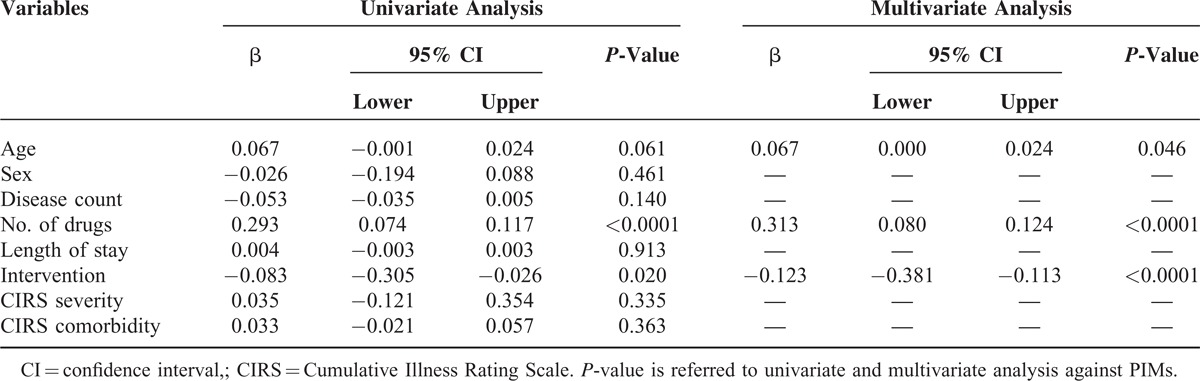
Linear Regression Analysis for Potentially Inappropriate Medications (PIMs)

**TABLE 5:**
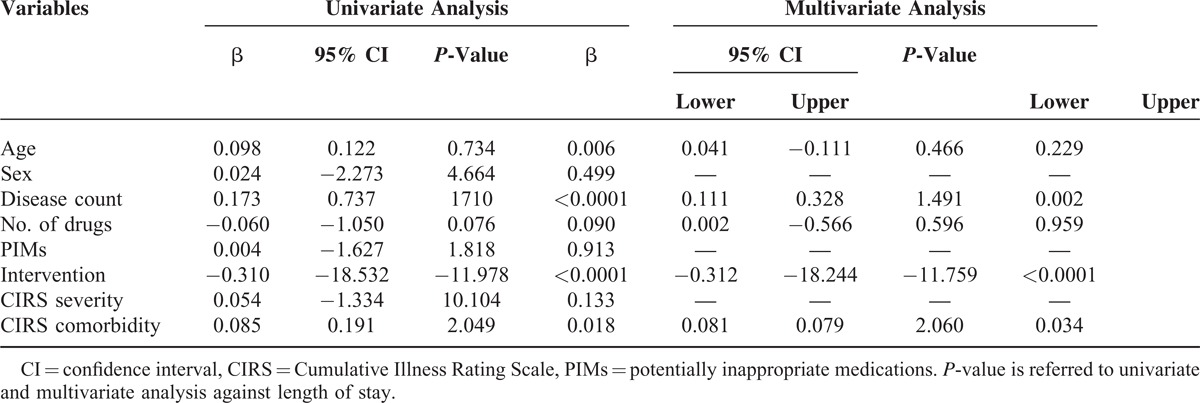
Linear Regression Analysis for Length of Stay

## DISCUSSION

In our study, the used educative instrument was able to significantly reduce the number of PIMs (Figure 2), and the length of hospitalization, suggesting as this tool could be very helpful especially in complicated patients with several comorbidities and drugs.

Several studies showed that PIMs are common in ambulatory settings, in nursing homes and in Emergency departments, and that inappropriate therapy is associated to increased Adverse Drugs Reactions, morbidity, mortality, and resource use.^9^ The optimization of drug prescription is thus becoming an objective necessity of health systems.^22^ Many studies based on the use of educative interventions demonstrated their efficacy in reducing the inappropriate prescriptions. Avorn et al^23^ examined the impact of an academic detailing intervention consisting in a clinical education of the medical staff in the principles of geriatric psychopharmacology on the use of psychotropic medications in nursing home residents. Similar to our study, inappropriateness scores for use of psychoactive drugs declined significantly in the intervention nursing homes compared with those from the control. Mattison et al^24^ also conducted a study to determine whether a computerized provider system could decrease orders for potentially inappropriate medications as defined by a subset of Beers list medications in hospitalized older patients. The authors concluded that specific alerts embedded into a computer-decision system, used in patients over 65 years, can decrease the number of inappropriate prescriptions quickly and specifically. However, the generalizability of most of these studies is limited because the investigators developed their own software programs to conduct the intervention that are not available to the public.^25^ Recently it has been demonstrated that computer-based systems can be implemented as decision resources, similar to the system found to be effective by Mattison et al^24^ for instance as a pop-up window in electronic medical records systems.^26^

In our study, the use of the tool was able to significantly reduce the length of hospital stay (Table 4), a well-known risk factor for mortality and complications.^26–28^ In particular, hospital length of stay is a potentially useful measurement of morbidity and a major determinant of the cost of medical care.^28^ Therefore, therapy should be guided by the appropriateness that is accomplished through the evaluation of the risk/benefit ratio, essentially when the potential benefits of a drug outweigh the potential risks. Starting from the consideration that the problems related to drug intake include factors related to the patient (emotional factors, simple forgetfulness, lack of training/information) and the doctor (deficit of information to the patient, complex therapies, bad doctor/patient relationship), it is necessary to develop strategies involving patients, families, caregivers, and family physicians engaged in an essential role in the correct medication, in the reduction of iatrogenic damage and increasing the adhesion.^29–31,32^

### Limitations

A limitation of our study could be considered the absence of ADRs as primary endpoint. The reason of this lack is that ADRs are objective of a greater study that our group is performing. An additional limitation could be represented by the use of Beers 2003 instead of 2012 criteria. This lack was related to the starting date of the project that was settled before the Beers 2012 criteria publication. However, in looking at the updated “drugs-to-avoid” list, it is important to note that of those therapeutic classes/medications removed from the previous criteria, 7 were due to drugs being withdrawn from the market since the last time the criteria were published in 2003 (eg, propoxyphene) and 1 removal was due to the lack of evidence that long-term stimulant laxatives lose effectiveness or result in unacceptable adverse effects.^32^ Then, the recommendation NOT to use >325 mg per day of ferrous sulfate to treat iron deficiency anemia (that represented 1 of the major causes of PIM in our study) applies to adults of all ages and thus was dropped.^33,34^

## CONCLUSIONS

Controlled studies articulated through the use of “tools” computing should be planned in order to give a correct answer to the increasing need of prescription appropriateness in polypathology elderly patients who take several different drug treatments. On this basis, these data suggest that our educative/informative instrument may be useful in reducing the number of inappropriate prescriptions and length of hospitalization, especially in more severely ill patients and with a high number of used drugs, representing, therefore, an important approach in the management of elderly patients characterized by polypharmacotherapy and comorbidity.
